# Analysis of the Genetic Comorbid Mechanisms of Type 2 Diabetes, Alzheimer′s Disease, and Hypertension Using Network Modularization

**DOI:** 10.1155/bmri/8877510

**Published:** 2026-02-18

**Authors:** Siwei Tian, Wenjing Zong, Ziling Zeng, Jingai Wang, Qikai Niu, Siqi Zhang, Huamin Zhang, Bing Li

**Affiliations:** ^1^ Institute of Chinese Materia Medica, China Academy of Chinese Medical Sciences, Beijing, China, cacms.ac.cn; ^2^ Data Center of Traditional Chinese Medicine, China Academy of Chinese Medical Sciences, Beijing, China, cacms.ac.cn; ^3^ Institute of Basic Theory for Chinese Medicine, China Academy of Chinese Medical Sciences, Beijing, China, cacms.ac.cn

**Keywords:** Alzheimer′s disease, hypertension, molecular mechanism, network module, Type 2 diabetes

## Abstract

**Background:**

Type 2 diabetes mellitus (T2DM), Alzheimer′s disease (AD), and hypertension (HTN) tend to be comorbidities and mutually influence each other; however, the mechanisms underlying their association remain unclear. This study was aimed at identifying genes associated with susceptibility to these three diseases and their mechanisms of action using integrated network modularization analysis.

**Methods:**

The transcriptome data of T2DM, AD, and HTN were downloaded from the GEO database to identify the differentially expressed genes (DEGs), and the coexpression modules of each disease were detected by WGCNA. *Z*_summary_ algorithm was used to identify the common modules of three diseases, and the driver genes of their comorbidity were identified by flow centrality (FC) and shortest distance indexes. Gene set enrichment analysis (GSEA) was performed to define the biological functions and pathways for each module and driver genes. The HPA database and CIBERSORT method were used to analyze the mechanisms of the shared key genes from the types of single cells and immune infiltration analysis.

**Results:**

Based on the 343 overlapping DEGs that were identified, four common modules between AD, T2DM, and HTN were identified using *Z*_summary_. GSEA revealed that the DEGs were mainly involved in the MAPK and mTOR signaling pathways. Eight key genes (ACTN4, BGN, PRELP, TSFM, UBC, ELAVL1, NRF1, and SUMO2) related to the comorbidities AD, T2DM, and HTN were identified by integrating the shared genes at the levels of DEGs, common modules, and FC‐based driver genes. As potential biomarkers, the expression of these key genes was significantly different between the three disease groups, and they were mainly expressed in endothelial cells, Langerhans cells, smooth muscle cells, and T cells. Immune infiltration analysis revealed that five different types of immune cells were related to these three diseases, including T‐regs and nonclassical monocytes.

**Conclusions:**

Common modules between T2DM, AD, and HTN and eight key susceptibility genes were identified, which may reflect the underlying mechanism of the comorbidity of T2DM, AD, and HTN. These results provide insights for the development of clinical therapies for these diseases.

## 1. Background

Type 2 diabetes mellitus (T2DM), Alzheimer′s disease (AD), and hypertension (HTN) tend to be comorbid and mutually influence each other, imposing a heavy burden on human health. However, the mechanisms shared between them remain unknown.

Network medicine employs network analysis methods and disease modules to elucidate complex disease associations, thereby facilitating the discovery of shared biological mechanisms underlying diseases. Complex diseases arise from the interplay of multiple molecular processes, wherein disease genes form tightly interconnected disease modules through interactions. These modules play pivotal roles in disease relationships [1]. With advances in bioinformatics and systems biology, network module approaches have matured within the field of traditional Chinese medicine research. Network modularity approaches treat disease‐associated biomolecules as network nodes, with molecular interactions serving as connecting edges, thereby constructing intricate biomolecular networks. Module‐based strategies are gaining increasing prominence over single‐gene and targeted therapies, proving crucial for elucidating the relationship between polygenic interactions and disease mechanisms. This provides both technical support and a research paradigm for deciphering the pathophysiological mechanisms underlying disease comorbidity [2–4].

AD is a progressive degenerative disease of the central nervous system, and its onset can go undetected. AD has been identified as an important public health issue by the World Health Organization (WHO) [5–8]. The main pathological features of AD are amyloid plaques and neurofibrillary tangles (NFTs). In addition, damage to nerve fibers, malnourished neurites, astrocyte proliferation, and microglial activation also occur, often alongside cerebral amyloid angiopathy [9–12]. Epidemiological surveys have shown that the number of patients with T2DM, a common disease in clinical settings, has increased worldwide in recent years [13–15]. The main causes are genetic and environmental factors, such as lifestyle factors, poor diet, and insufficient physical activity [16–19]. Typical symptoms include increased drinking, eating, and urinating and losing weight, particularly in a short amount of time. The main pathological characteristics are insulin resistance, defective *β*‐cell function, abnormal *α*‐cell function in pancreatic islets, and defective incretin secretion [20, 21]. HTN is a phenomenon in which the blood vessel wall pressure is higher than normal due to increased blood flow [22–24]. Typical symptoms include headaches, fatigue, discomfort, arrhythmia, palpitations, and tinnitus. A recent survey showed that the prevalence of HTN among adults in China is increasing annually [25]. The causes of HTN are not fully understood, but the main causes are genetic factors and an unhealthy lifestyle [26, 27]. Immune mechanisms are an integral part of the multifactorial etiology of HTN and related organ damage [28–30]. The pathogenesis of HTN involves vascular, neural, and hormonal mechanisms as well as insulin resistance [31, 32].

AD is considered to be caused by a combination of genetic and environmental factors. T2DM is the most common endocrine and metabolic disease worldwide and is considered an independent risk factor for cognitive dysfunction and dementia, and it is closely related to AD [33–35]. T2DM is associated with an increased risk of AD, which is related to changes in insulin levels, vascular damage, and functional neuron damage, similar to the mechanisms involved in HTN [36–38]. Therefore, it is important to identify whether there are associations between genetic susceptibility to AD, T2DM, and HTN [39, 40] and to identify the common pathogenesis between the three diseases. Moreover, there is a lack of studies on associations between AD, T2DM, and HTN [41]. In addition, with the development of bioinformatics and the application of gene chips, new methods and ideas have been developed for such analyses in the medical field [42]. Common transcriptional patterns offer new perspectives on the shared etiology of different diseases [43–45].

To find the key genes and molecular mechanisms shared between these three diseases, in this study, we obtained microarray datasets of AD, HTN, and T2DM from the Gene Expression Omnibus (GEO) database and screened for common differentially expressed genes (DEGs) between the three diseases. Based on these DEGs, we used gene set enrichment analysis (GSEA) to identify the relevant biological functions. Next, we connected the modules using weighted gene coexpression network analysis (WGCNA) combined with external clinical features. Using the *Z*_summary_ method, shared genes were identified at the module level. Then, driver genes were identified using flow centrality (FC) and shortest distance analysis. Finally, we further verified intersecting genes among the DEGs, common modules, and driver genes as the key genes. Additionally, we explored the cell types associated with the key genes and discovered possible common pathogenic mechanisms in the three diseases.

## 2. Methods

### 2.1. Data Sources

The datasets used in this study, including the AD dataset GSE16759, T2DM dataset GSE55650, and HTN dataset GSE28345, obtained from the National Center for Biotechnology Information (NCBI) Gene Expression Omnibus (GEO) database (http://www.ncbi.nlm.nih.gov/geo/) [46]. The datasets were filtered, background‐corrected, log2 transformed, and normalized. The Limma package [47] was used to compare the data from the normal and disease groups, and the screening criteria for the DEGs were |log2 fold change| > 1 and *p* < 0.05. The common downregulated DEGs obtained from the AD, T2DM, and HTN datasets were visualized using a volcano map.

### 2.2. Functional Enrichment Analysis

The Metascape database (http://www.metascape.org) was used to annotate and visualize the common DEGs between the disease groups. Biological process (BP), molecular function (MF), cellular component (CC), and Kyoto Genome Encyclopedia (KEGG) [48, 49] functional enrichment analyses were conducted to identify the biological functions and signaling pathways related to the DEGs (count = 2; EASE = 0.01; and species = *Homo sapiens*), and DEGs with *p* < 0.05 were considered significant [50].

### 2.3. GSEA of the Key Genes

GSEA was conducted [51] using GSEA Software 3.0 (http://software.broadinstitute.org/gsea/index.jsp). The samples were divided into two groups according to the disease datasets. We downloaded subsets from the Molecular Signatures database to identify relevant pathways and molecular mechanisms. Based on the gene expression profiles and phenotype grouping, the following characteristics were considered significant: a minimum gene set of 5, a maximum gene set of 5000 and 1000 resamplings, *p* < 0.05, and an absolute value of an NES greater than or equal to 1.

In this study, we performed GSEA of the DEGs between the disease and control groups. GO and KEGG analyses of the three diseases were conducted by grouping the conditions and sample expression data of AD, T2DM, and HTN. Then, BP, CC, and MF functional enrichment analyses were performed to identify the pathways and biological functions shared between the three diseases.

### 2.4. Co‐expression Module Identification and Associated Clinical Traits

WGCNA [52] was employed to analyze the GSE16759, GSE55650, and GSE28345 datasets to determine the gene modules associated with AD, T2DM, and HTN. We removed the absent data points before clustering, and the mean values were used for duplicate gene calculations. Following the scale‐free topology criterion, a “soft” threshold power (*β*) was established. Subsequently, a biologically significant, scale‐free network was constructed. In addition, a topological overlap matrix (TOM) was derived from the adjacency matrix using a dynamic tree‐cutting algorithm to identify gene modules. Then, the gene signature (GS), module membership (MM), and modules correlated with clinical characteristics were computed, culminating in the visualization of the feature gene network. To identify the pivotal modules associated with AD, T2DM, and HTN, the correlation coefficients and *p*‐values between the expression levels of each module and the clinical traits were analyzed. Finally, the significant module genes from the WGCNA of AD, T2DM, and HTN were combined and visualized using Venn diagrams to identify shared genes.

### 2.5. Analysis of Common Modules Using *Z*_*s* *u* *m* *m* *a* *r* *y*_ Statistic

To identify the common modules between AD, T2DM, and HTN, quantitative analysis was performed using the *Z*_summary_ method [53, 54]. Pairwise comparisons of modules associated with the three diseases were performed, and the modules with *Z*_summary_ ≥ 2 were considered to be common modules among the three diseases. Then, we identified intersecting genes at the common module level and common genes at the DEG level to obtain the shared genes.

### 2.6. Screening of the Driver Genes of AD, T2DM, and HTN

To screen the different driver genes between the disease modules of AD, T2DM, and HTN, the FC [55] and the shortest distance [56] were calculated. The driver genes for the three diseases were screened using the maximum FC and minimum shortest distance values. The maximum FC value and minimum shortest distance value were used as the standards.

The shortest distance is the sum of the distances between all nodes in *A* and *B* and is normalized by the product of their sizes using the following formula:

dIAIBshortest=1IA×IB∑a∈IA,b∈IBdia,b

where *I*(*A*) is the set of components in module *A* and *d* *i*(*a*, *b*) is the distance between two component nodes in modules *a* and *b*.

The FC value of a specific node is defined as follows:

FCA,Bv=1AB∑a∈A,b∈Bσabvσab

where *A* and *B* represent the different disease modules, *σ*_*a* *b*_(*v*) is the number of shortest paths from *A*|or|*B* passing through node *v*, *σ*_ab is the total number of shortest paths from *a* to *b*, and | *A* | or | *B* | is the size of the set.

In this study, we calculated the shortest distance and FC between pairwise genes among the AD, T2DM, and HTN datasets. Based on the screening criteria (the maximum, FC_max,_ and the shortest distance, min), the shortest distance and FC values of the Top 10 genes for each disease were calculated. Finally, the genes associated with all three diseases were considered to be common driver genes.

### 2.7. Identification of Key Genes

The previously identified shared genes among patients with AD, T2DM, and HTN at the DEG level and the shared genes in the common modules were further analyzed. Moreover, the intersecting shared genes obtained at the DEGs and module levels were obtained. To comprehensively and accurately identify the key genes, we also selected driver genes using shortest distance and FC screening.

To sum up, in this study, we took the intersection genes of DEGs hierarchy and module hierarchy, as well as the driver genes as the key genes, which were for subsequent study analysis.

### 2.8. Validation of the Expression of Key Genes Using the Human Protein Atlas (HPA) Database

The HPA database [57] contains information on RNA and protein expression in different human tissues and organs that has been obtained using transcriptomic and proteomic techniques. In this study, the expression of key genes and recognized cell‐type markers in distinct single‐cell‐type clusters was obtained from the HPA database. The levels of different single‐cell types were sorted according to the normalized tags per million (nTPM), and the cell types related to the key genes were determined.

### 2.9. Immune Infiltration Analysis of Key Genes

The CIBERSORT deconvolution algorithm [58] is a machine learning method based on linear support vector regression, which can be used to calculate the abundance of 22 types of immune cells in tissues or organs. Our analysis was conducted in R, and the CIBERSORT deconvolution method was used to simulate the transcriptional characteristic matrix of 22 types of immune cells, including B cells, plasma cells, T cells, natural killer cells, monocytes, macrophages, dendritic cells, mast cells, eosinophils, and neutrophils. We compared the immune cell infiltration levels of peripheral blood mononuclear cell (PBMC) samples from each disease group with those of the normal samples. Moreover, the relationships between key genes and immune cells in AD, T2DM, and HTN were explored.

## 3. Results

### 3.1. Screening of DEGs Associated With AD, T2DM, and HTN

As shown in Figures 1a, 1b, and 1c, the numbers of up‐ and downregulated genes associated with AD, T2DM, and HTN were determined. A total of 2174 genes (upregulated: 914; downregulated: 1260) were identified in the AD datasets; 884 genes (upregulated: 506; downregulated: 378) were identified in the HTN datasets, and 2675 genes (upregulated: 1254; downregulated: 1421) were identified in the T2DM datasets. In addition, by analyzing the intersecting upregulated and downregulated genes associated with the three diseases, we identified the following 13 overlapping genes among AD, T2DM, and HTN: ARL17B, AGTRAP, PRPF4, TIMP2, TPP1, TSFM, C1orf21, DHRS7, PRELP, BGN, PARP1, ACTN4, and LACC1 (Figure 1d).

Figure 1Identification of shared differential genes. (a–c) Volcano map of DEGs in AD, T2DM, and HTN. Blue indicates downregulated DEGs, and red indicates upregulated DEGs. (d) Venn diagram displaying co‐upregulated and co‐downregulated DEGs. (e, f) GO and KEGG analyses of DEGs based on the Metascape database.

**Figure:**
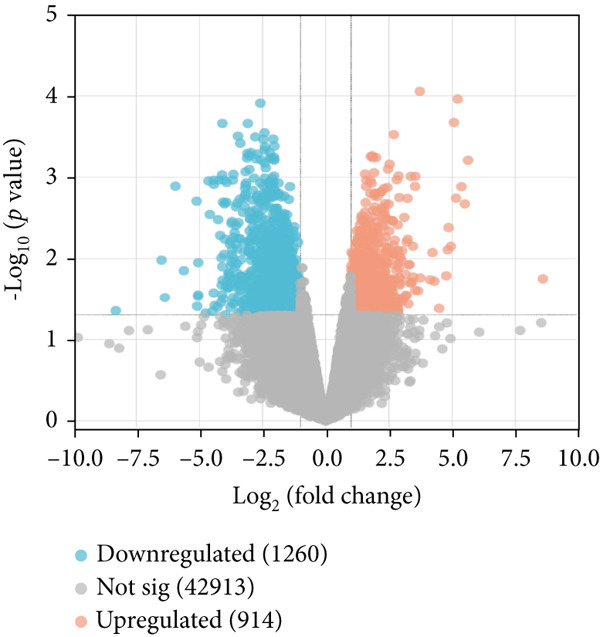
(a)

**Figure:**
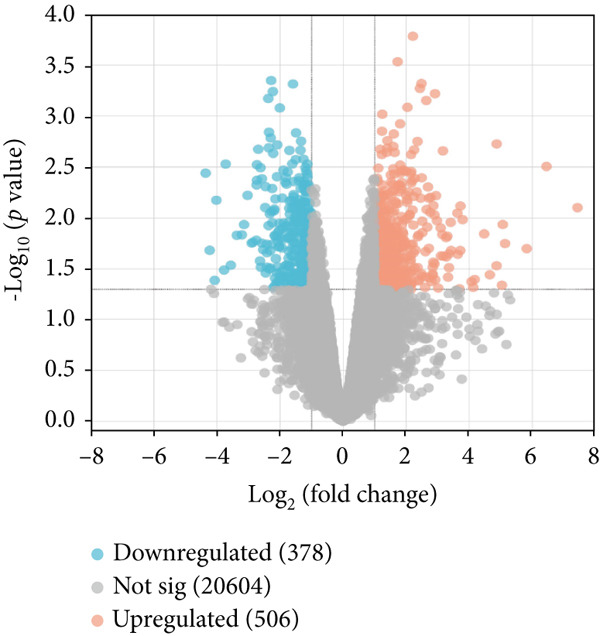
(b)

**Figure:**
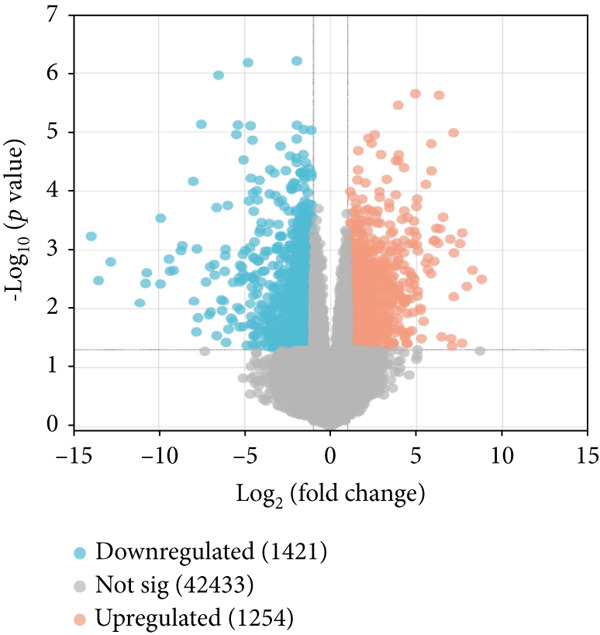
(c)

**Figure:**
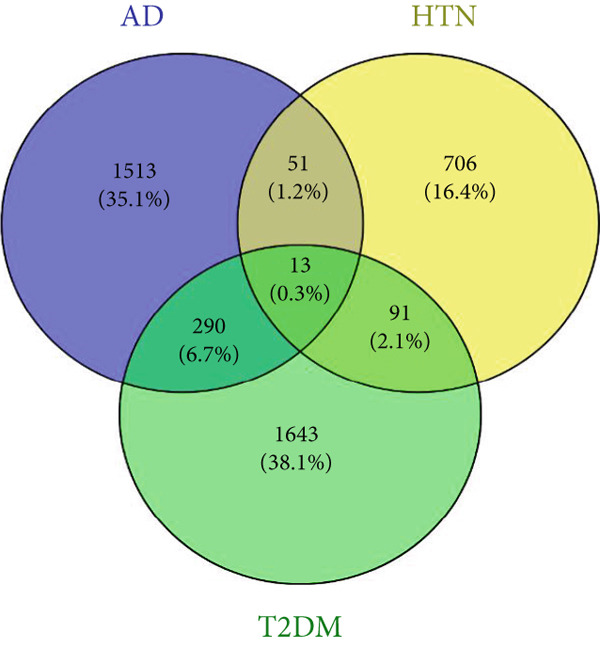
(d)

**Figure:**
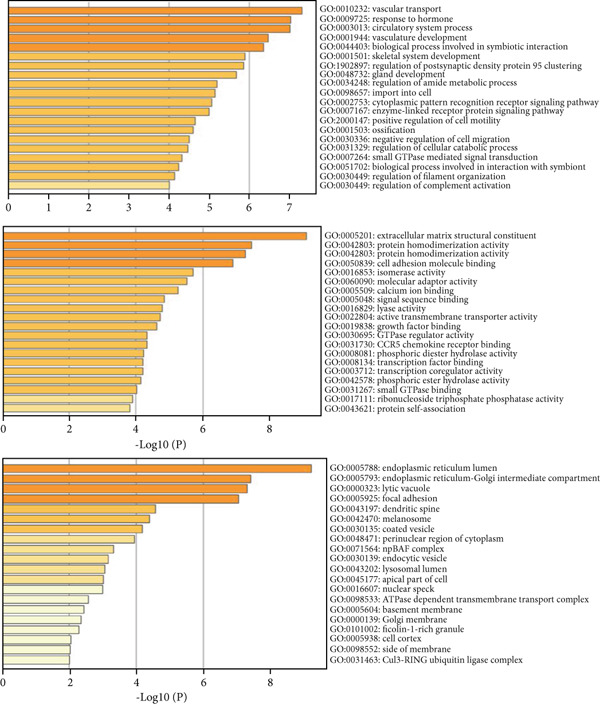
(e)

**Figure:**
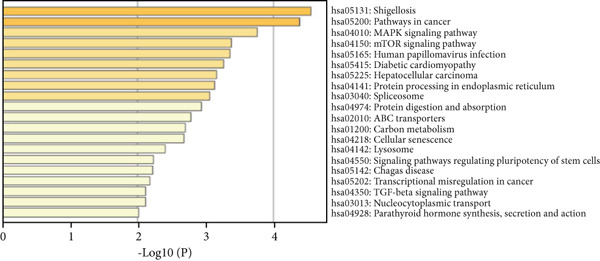
(f)

### 3.2. Functional and Pathway Enrichment Analyses

We screened the DEGs related to AD, T2DM, and HTN according to the screening criteria (| log2 fold change | > 1 and *p* < 0.05). Subsequently, we identified 343 shared genes among the three diseases. The GO terms enriched in the 343 shared genes between AD, T2DM, and HTN are illustrated in Figure 1e.

To determine the biological functions and pathways associated with the identified overlapping DEGs, we performed GO and KEGG analyses, and the results were plotted using bar diagrams and net plots. Based on the GO analysis results, we found that the shared DEGs between the three diseases were enriched in the BP terms “vascular transport,” “response to hormones,” and “circulatory system process.” For the MFs, the enriched terms were “extracellular matrix structural constituent,” “protein domain specific binding,” and “protein homodimerization activity.” The CC terms “endoplasmic reticulum lumen,” “endoplasmic reticulum‐Golgi intermediate compartment,” and “lytic vacuole” were enriched (Figure 1e). KEGG analysis showed that the shared DEGs were mainly enriched in the MAPK and mTOR signaling pathways (Figure 1f).

### 3.3. GSEA of Biological Functions and Pathways Associated With Key Genes

Based on the clinical groupings (AD, T2D, and HTN) two subgroups were formed: the disease and control groups. We conducted GSEA of data from patients with one of the three diseases. Using certain screening criteria, we identified and ranked the biological functions and pathways with statistically significant differences in their activity between the two subgroups.

The results of the GO analysis revealed significant differences in BP, CC, MF, and KEGG enrichment terms between these two subgroups. As shown in Figure 2, “viral gene expression,” “response to reactive oxygen species,” “cellular response to chemical stress,” and “mitochondrial matrix” were enriched among the BP terms. “endoplasmic reticulum–golgi intermediate compartment,” “neuron to neuron synapse,” and “transporter complex” were enriched among the CC terms. With regard to the MF terms, “receptor regulator activity,” “translation factor activity RNA binding,” and “magnesium ion binding” were enriched. The main enriched KEGG terms were “endocytosis” and “insulin signaling pathway.”

Figure 2GSEA of key genes in the disease and control groups. GO‐BP, GO‐MF, GO‐CC, and KEGG enrichment terms in the (a) AD, (b) T2DM, and (c) HTN groups.

**Figure:**
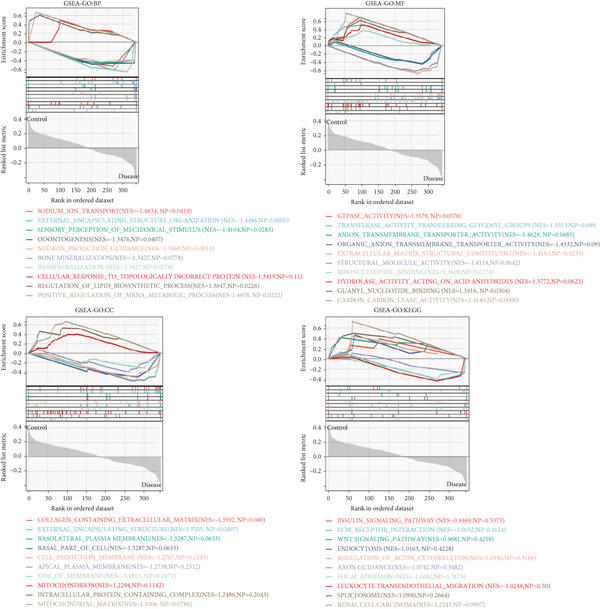
(a)

**Figure:**
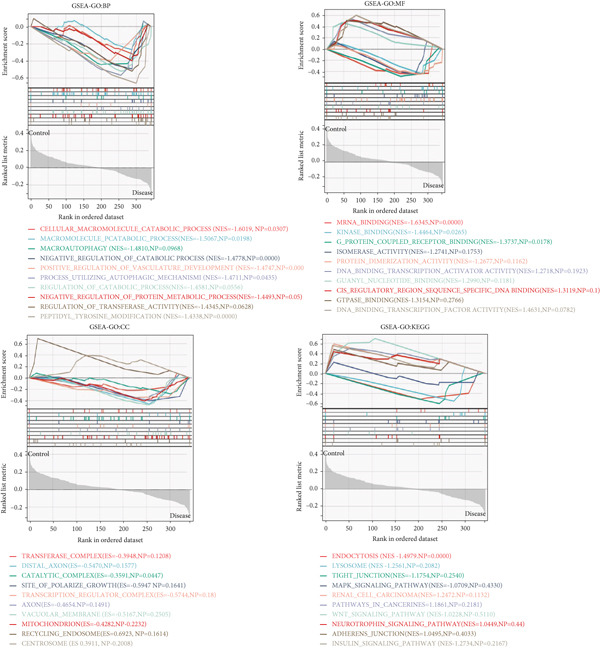
(b)

**Figure:**
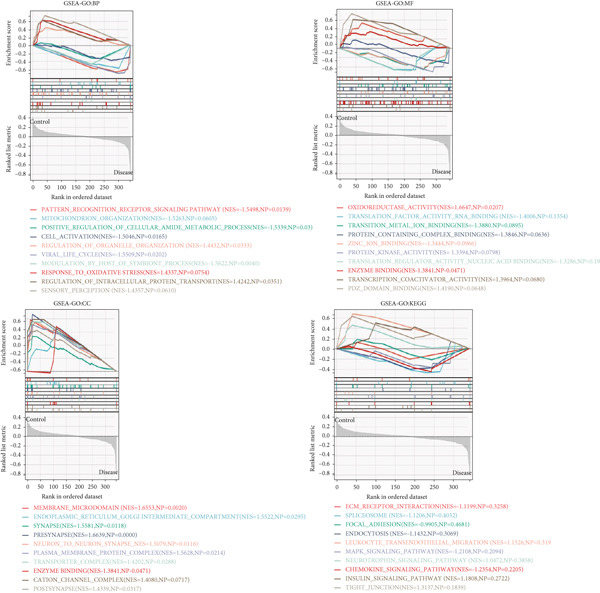
(c)

### 3.4. Identification of Coexpressed Modules Associated With AD, T2DM, and HTN

As shown in Figures 3a, 3d, and 3g, WGCNA of the GSE16759 datasets resulted in the identification of 20 modules associated with AD. The associations between each module and AD were visualized using a heat map and Spearman′s correlation coefficient. The magenta, blue, and brown modules were positively correlated with AD (r = 0.77, 0.76, and 0.76, respectively; *p* = 0.03), whereas the light green and turquoise modules were negatively correlated with AD (*r* = −0.90 and −0.78, respectively; *p* = 0.002 or 0.02). These were identified as the key modules associated with AD.

Figure 3Identification of co‐expressed gene modules and their correlation with the three diseases. (a–c) Analysis of network topology for various soft‐thresholding powers of AD, HTN, and T2DM. (d–f) Cluster dendrogram of the 343 shared DEGs based on the topological overlap. Each branch of the cluster tree with a certain color represents a coexpression module; AD, HTN, and T2DM are shown from left to right. (g–i) Heatmap of the module–trait relationships of the three diseases. The row–column intersection shows the *p* values that correspond to each other. Shown from left to right: AD, HTN, and T2DM.

**Figure:**
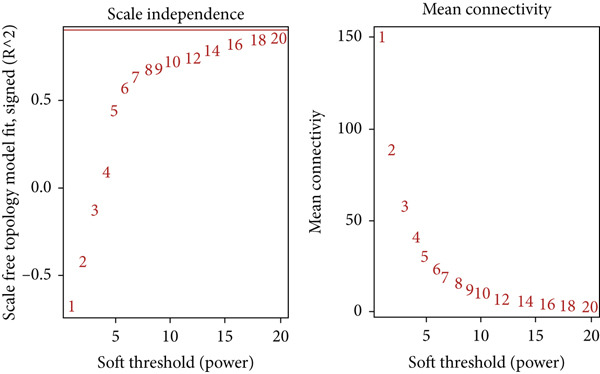
(a)

**Figure:**
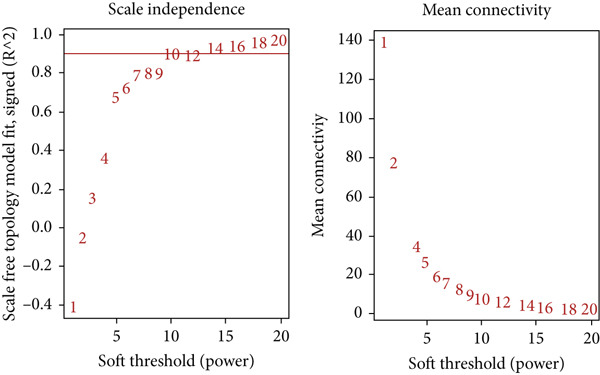
(b)

**Figure:**
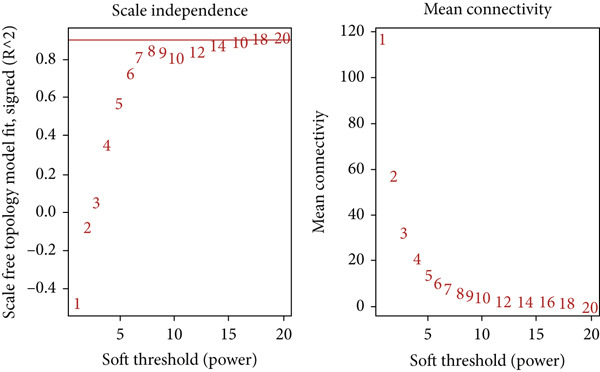
(c)

**Figure:**
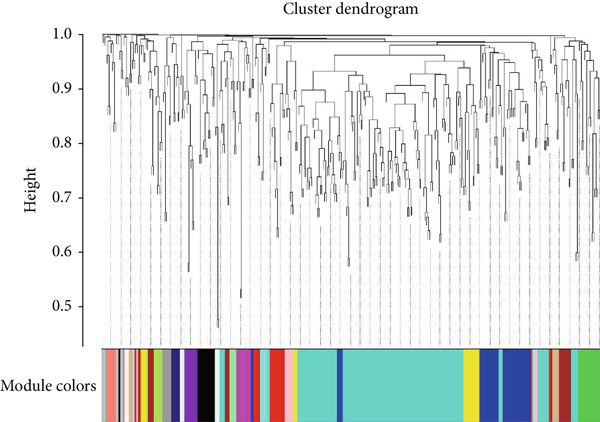
(d)

**Figure:**
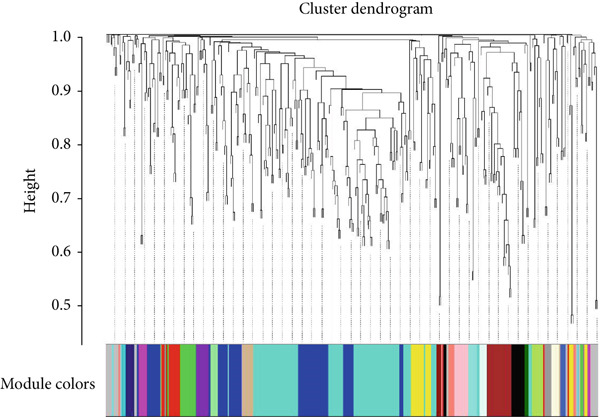
(e)

**Figure:**
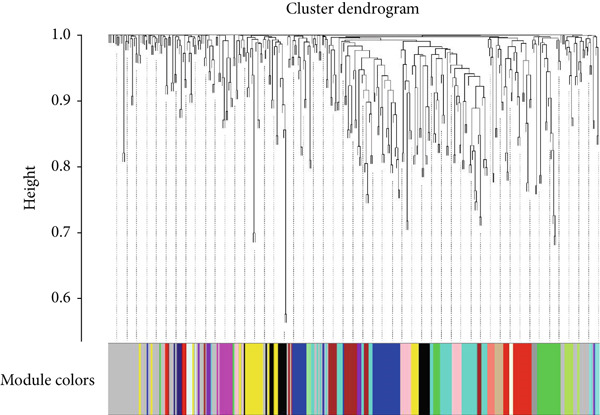
(f)

**Figure:**
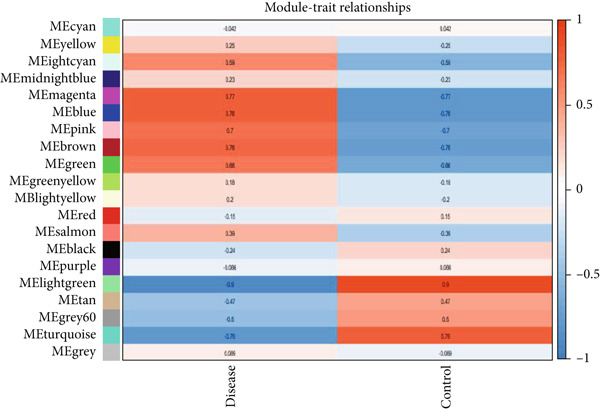
(g)

**Figure:**
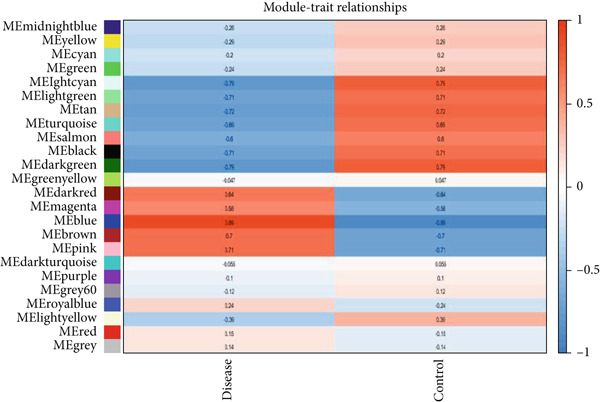
(h)

**Figure:**
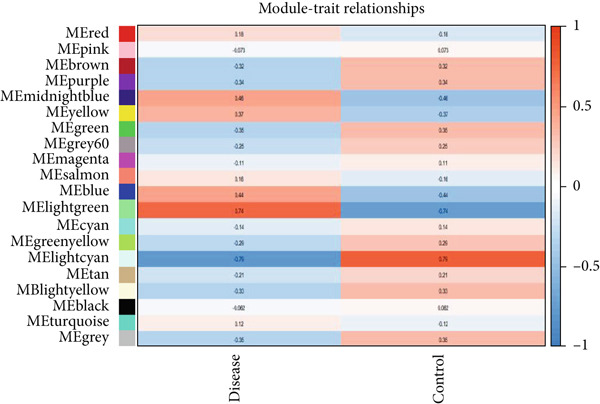
(i)

As shown in Figure 3b, 3e, 3h, WGCNA of the GSE28345 dataset resulted in the identification of 24 modules associated with HTN. Analysis of the associations between the gene modules and the disease and control HTN datasets revealed that the blue, brown, and pink modules were positively correlated with HTN (*r* = 0.89, 0.70, and 0.71, respectively; *p* = 0.003, 0.05, and 0.05, respectively). There were five negatively correlated modules (including light green and light cyan), which were identified as differential modules between the disease and control groups.

Similarly, as shown in Figures 3c, 3f, and 3i, WGCNA of the GSE55650 dataset resulted in the identification of 20 modules associated with T2DM. The light green module was positively correlated with T2DM (*r* = 0.74, *p* = 0.006), and the light cyan module was negatively correlated (*r* = −0.79, *p* = 0.002) with T2DM.

### 3.5. Common Modules and the Related Genes Shared Between AD, T2DM, and HTN

As shown in Figure 4, to screen for the common modules between AD, T2DM, and HTN, we conducted a pairwise comparative analysis of genes associated with the three diseases using *Z*_summary_. We considered modules with *Z*_summary_ ≥ 2 as shared by two diseases. As shown in Figures 4a, 4b, 4c, 4d, 4e, and 4f. For AD, two modules that were shared by HTN and T2DM were identified. For HTN, one common module was identified in the comparisons with AD and T2DM. For T2DM, there were two common modules shared with AD, and one common module with HTN. We identified four common modules between the three diseases, and shared genes at the level of the common modules and DEGs were obtained. As shown in Figure 4g, the shared genes at the module and gene levels intersected to yield four shared genes, and their names are shown in Table 1.

Figure 4The shared modules between AD, T2DM, and HTN. (a–f) The shared modules identified through pairwise comparative analysis of the three diseases using *Z*_summary_. The *x*‐axis displays module size, and the *y*‐axis displays *Z*_summary_ values. Each labeled color represents a module. (g) Venn diagram of the intersection of shared genes at the module and gene levels. The shared genes are listed in Table 1.

**Figure:**
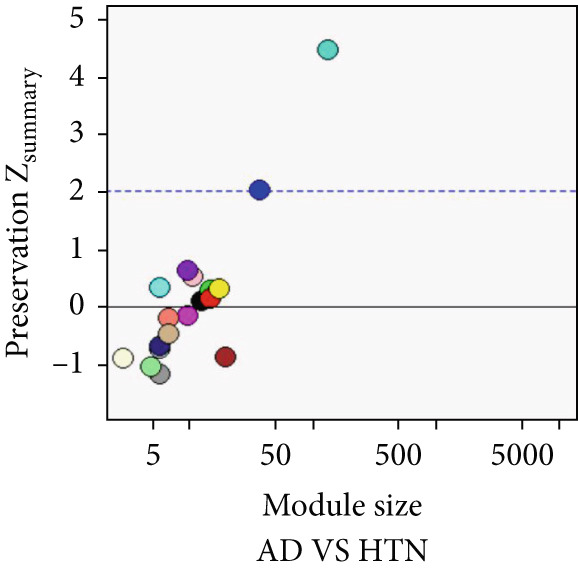
(a)

**Figure:**
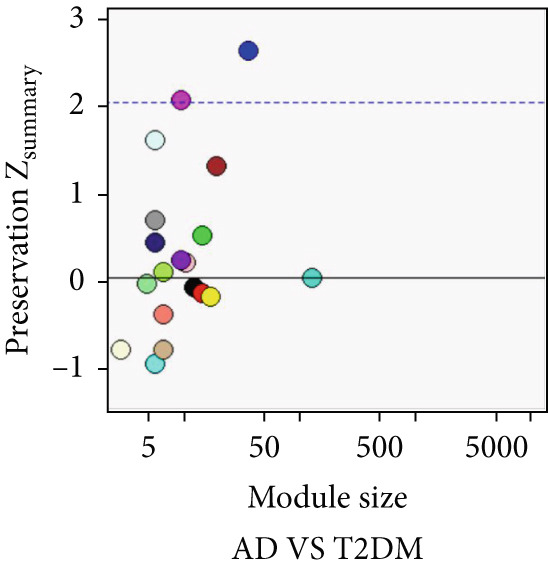
(b)

**Figure:**
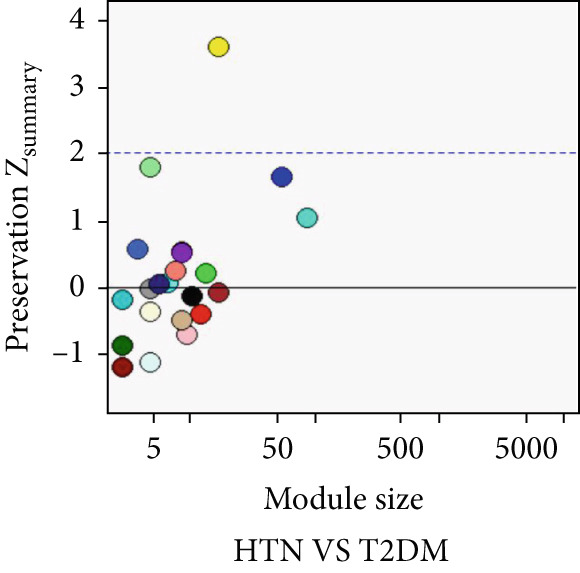
(c)

**Figure:**
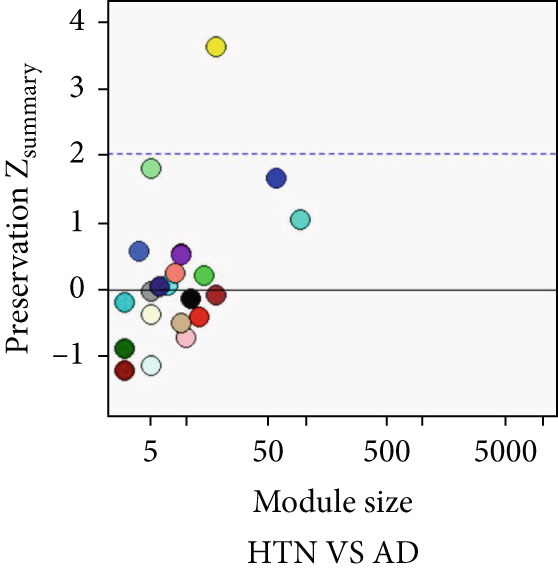
(d)

**Figure:**
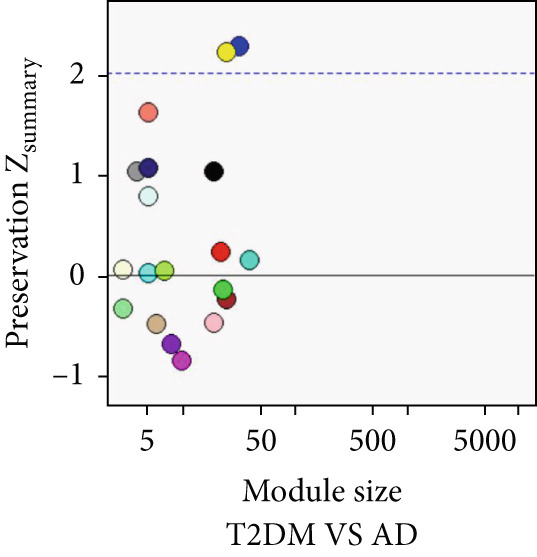
(e)

**Figure:**
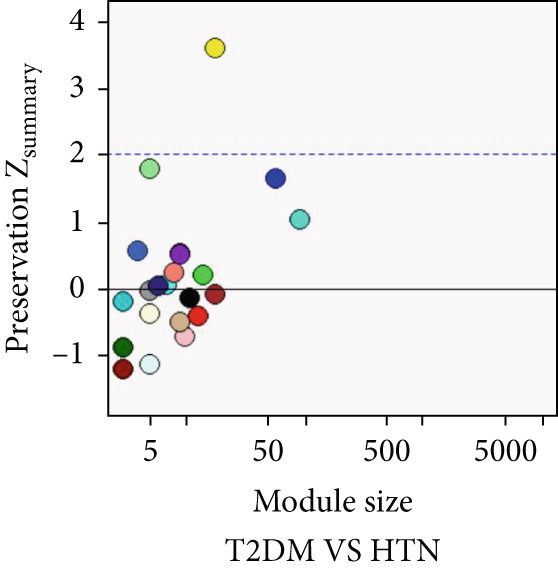
(f)

**Figure:**
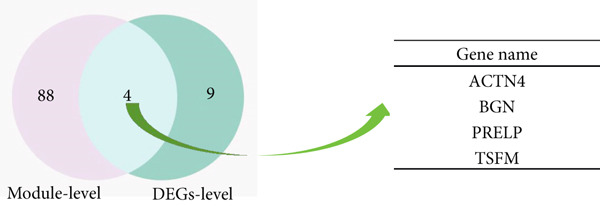
(g)

**Table 1:** The identified driver genes associated with AD, T2DM, and HTN.

**Disease**	**FC-based driver genes**	**Shortest distance-based driver genes**
AD VS HTN	UBC (6224.35)	UBC (320)
	ELAVL1 (450.42)	ELAVL1 (459)
	APP (329.73)	APP (467)
	NRF1 (300.68)	NRF1 (472)
	FN1 (256.29)	JUN (474)
	CIB1 (243.04)	FN1 (479)
	TP53 (220.33)	SUMO2 (480)
	MYC (164.46)	CUL3 (484)
	COL1A2 (142.01)	GRB2 (486)
	CUL3 (138.87)	YWHAZ (492)

AD VS HTN	UBC (240.10)	UBC (247)
	CIB1 (187)	ELAVL1 (355)
	NRF1 (40.63)	APP (358)
	SUMO2 (12.46)	NRF1 (363)
	SMAD2 (7.41)	JUN (365)
	PRKDC (5.72)	SUMO2 (369)
	SUMO1 (5.33)	FN1 (371)
	TCTN3 (5.17)	CUL3 (376)
	APP (4.16)	GRB2 (380)
	MAPK1 (3.89)	YWHAZ (381)

HTN VS T2DM	UBC (73.25)	UBC (79)
	CIB1 (58)	ELAVL1 (114)
	NRF1 (11.16)	NRF1 (115)
	SUMO2 (3.38)	GRB2 (116)
	PRKDC (3.25)	FN1 (118)
	SMAD2 (2.51)	CUL3 (118)
	AURKB (2.12)	APP (119)
	TP53 (2)	JUN (119)
	SUMO1 (1.99)	SUMO2 (119)
	ELAVL1 (1.78)	MYC (120)

### 3.6. Driver Genes of AD, T2DM, and HTN

Among the genes clustered in the common modules, the FC and shortest distance methods were used to identify the driver genes of AD, T2DM, and HTN. Using screening criteria of FC_max_ and shortest distance (_min_), we analyzed the Top 10 key genes associated with the three diseases and identified four driver genes: UBC, ELAVL1, NRF1, and SUMO2.

### 3.7. Key Genes Associated With AD, T2DM, and HTN

Using the results described above, we merged the shared genes at the common module and DEG levels with the driver genes (Table 1). Among these, we screened eight common genes (ACTN4, BGN, PRELP, TSFM, UBC, ELAVL1, NRF1, and SUMO2) between AD, T2DM, and HTN, which we considered to be key genes and used these for subsequent analyses.

### 3.8. Expression Characteristics of the Candidate Diagnostic Biomarkers

This study was aimed at elucidating the significance of key genes in the onset of AD, T2DM, and HTN. Therefore, a comprehensive analysis of the expression profiles of the key genes in the patient cohort was conducted, and we compared them with those of the control group.

The results revealed the expression patterns of the key genes involved in AD, HTN, and T2DM. The expression of one key gene among the different disease groups was significantly different from that in the control group. The expression profiles of the key genes SUMO2, TSFM, UBC, and ELAVL1 in AD, HTN, and T2DM are shown in Figures 5a, 5b. 5c, and 5d. The expression profiles of the remaining key genes are shown in Figures S1, S2, S3, and S4.

Figure 5Expression profiles of four key genes in the control and disease groups. (a) SUMO2 expression, (b) TSFM expression, (c) UBC expression, and (d) ELAVL1 expression. Shown from left to right: AD, HTN, and T2DM.

**Figure:**
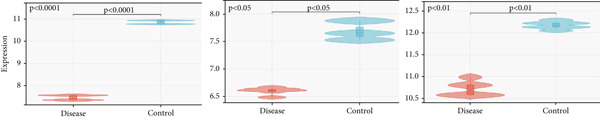
(a)

**Figure:**
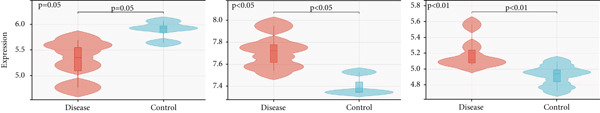
(b)

**Figure:**
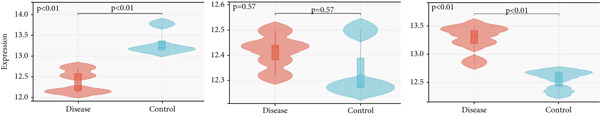
(c)

**Figure:**
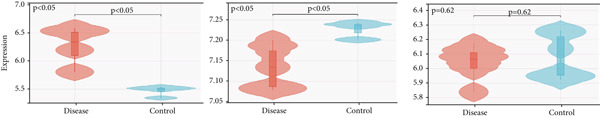
(d)

### 3.9. Cell‐Specific Enrichment and Expression Analyses of Key Genes

#### 3.9.1. Single‐Cell Analysis

Using single‐cell analysis of the HPA database, according to nTPM, we sorted the expression levels of the eight key genes in different single cell types and determined the gene expression of the Top 50 key genes as the intersecting genes.

Figure 6a shows the gene expression of the eight key genes in different types of single cells. The Top 50 cell types expressing the eight key genes were selected, and we found that the main single‐cell types related to AD, HTN, and T2DM were endothelial cells, Langerhans cells, smooth muscle cells, and T cells.

Figure 6Types of cells related to AD, HTN, and T2DM. (a) Single‐cell analysis of the HPA database based on the nTPMs of eight key genes. (b) Immune cell‐type analysis of the HPA database based on the nTPMs of eight key genes. (c–e) The immune cells present in the disease and control groups of AD, HTN, and T2DM.

**Figure:**
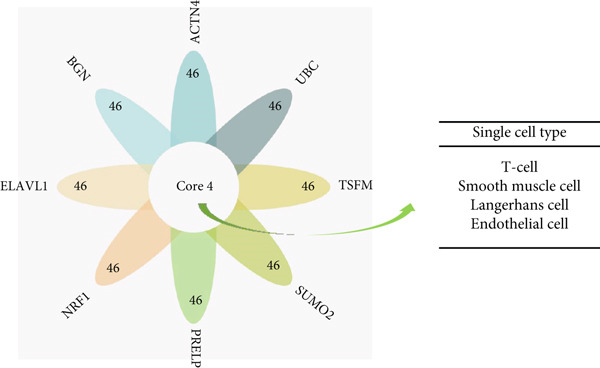
(a)

**Figure:**
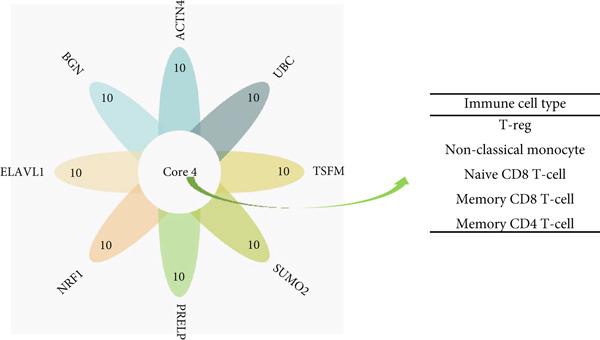
(b)

**Figure:**
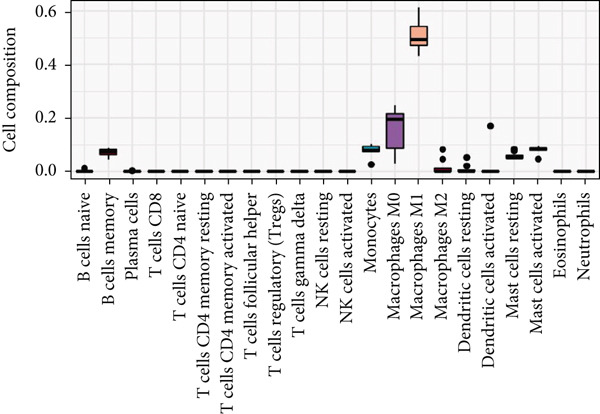
(c)

**Figure:**
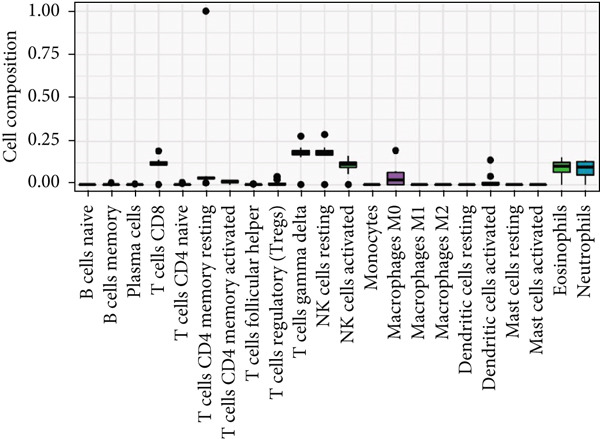
(d)

**Figure:**
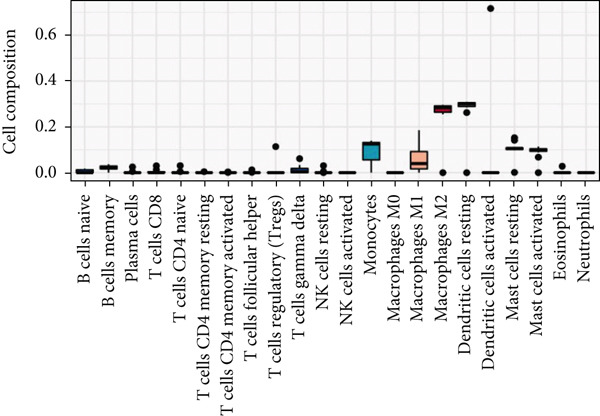
(e)

#### 3.9.2. Immune Infiltration Analysis at the Disease and Key Gene Levels

We explored the immune‐related cell types present in AD, HTN, and T2DM associated with the key genes using the HPA database. As shown in Figure 6b, according to the nTPM values, the Top 15 immune cell types expressing the eight key genes were selected. Five different types of immune cells were related to the three diseases, including T‐regs and nonclassical monocytes. For the analysis at the disease level, we used CIBERSORT to analyze the data of two subgroups, the disease and control groups. As shown in Figures 6c, 6d, and 6e, macrophages and monocytes were associated with AD, HTN, and T2DM.

## 4. Discussion

T2DM is closely related to the onset of AD, which is also an independent risk factor for the occurrence and development of T2DM and is mainly related to insulin levels, vascular damage, and functional neuron damage. HTN is associated with neural mechanisms, insulin levels, and blood vessels [39, 40]. However, research on the pathogeneses of AD, HTN, and T2DM is limited. Therefore, in this study, we screened for common biomarkers of AD, HTN, and T2DM to explore the common pathogeneses of these three diseases. This study screened shared genes across three diseases at two levels. Firstly, 343 DEGs were identified from the GEO dataset. Secondly, at the module level, codisease‐associated modules were identified using WGCNA combined with the *Z*_summary_ algorithm. By integrating fold change values and shortest distance metrics, eight key genes were ultimately selected. This approach accounts for synergistic regulatory interactions between genes while addressing the limitation that expression differences within modules may be non‐significant. By validating module‐specific expression abnormalities through DEGs and deciphering functional synergistic mechanisms via module structure analysis, this strategy achieves multilevel codisease mechanism elucidation from “differential expression” to “functional synergy.”

This study identified 343 shared genes among the three diseases by screening DEGs and enrichment of the terms associated with the shared genes through GO and KEGG analyses. The 92 genes contained in the common modules intersected with 13 common DEGs, which led us to identify four shared genes. Four driver genes were identified using FC and the shortest distance method. In this study, eight key genes were identified and used for further research.

Subsequently, we observed statistical differences in gene expression between the disease and control subgroups of the three diseases, as demonstrated by violin plots. To further determine the common pathogenesis of these three diseases, we analyzed the proportions of single and immune cell types.

In studies examining the comorbidity of T2DM, HTN, and AD, screening key genes and comparing them with prior research can reveal their regulatory value in co‐morbidity [59–62]. Previous studies indicate that UBC participates in tau protein ubiquitination in AD and pancreatic *β*‐cell apoptosis in T2DM. ELAVL1 promotes vascular remodelling in HTN by stabilising inflammatory factor mRNA. This study identifies both genes as possessing high FC values and shortest distances within the comorbidity network, functioning as pivotal nodes linking the pathological pathways of all three diseases and providing novel evidence for “multiple pathological linkages synergistically disrupted.” NRF1 is implicated in neuronal mitochondrial protection in AD and relates to insulin sensitivity in T2DM. SUMO2 primarily participates in vascular endothelial stress in HTN. This study suggests NRF1 regulates shared pathologies across the three diseases via antioxidant stress pathways, while SUMO2 forms a core biomarker combination with UBC and ELAVL1 for predicting comorbidity risk, with its expression significantly correlated with comorbidity progression. TSFM has previously been associated with skeletal muscle energy metabolism disorders in T2DM. BGN exhibits abnormal expression in HTN vascular remodeling and diabetic foot ulcers. WGCNA analysis revealed that the module containing TSFM was coenriched across all three diseases, with its dysregulation causing energy metabolism impairment. The module containing BGN not only participates in HTN vascular remodelling but also indirectly regulates T2DM blood supply and AD blood–brain barrier integrity, providing targets for module‐specific interventions. ACTN4 and PRELP have received limited attention in prior comorbidity studies. The former correlates with glomerular injury in T2DM nephropathy, while the latter has been implicated in AD brain matrix research. This study reveals their elevated expression in vascular endothelial/smooth muscle cells, with abnormal expression contributing to vascular‐related damage across all three conditions. This suggests they may play a significant role in vascular pathologies associated with comorbidity.

Regarding single‐cell types, four common cell types were enriched in AD, T2DM, and HTN: endothelial cells, Langerhans cells, smooth muscle cells, and T cells. Various roles of endothelial cells [63] in the three diseases have been observed previously: In AD, the brain capillary endothelial cell receptor plays an important role in the clearance of amyloid *β* (A*β*). Insulin resistance can affect vascular endothelial function, leading to the development of T2DM and HTN. Langerhans cells play an important role in antigen presentation and the initiation of immune responses. Currently, there is limited research on the relationship between Langerhans cells, HTN, and AD, with most studies focusing on inflammation, immune regulation, and immune responses. Langerhans cells play an important role in neuroinflammation and vascular inflammatory responses, and the occurrence of AD and HTN is closely related to inflammation. Moreover, abnormal protein deposition in the brain is a clinical feature of AD, and the role of Langerhans cells in antigen presentation may result in immune responses targeting abnormal proteins in the brain. The density of Langerhans cells in the corneal epithelium of patients with Type 2 diabetes is high, and the number of mature central corneal Langerhans cells increases with the aggravation of diabetic retinopathy [64].

The main immune cells associated with key gene expression were nonclassical monocytes, which help repair damaged blood vessels and participate in inflammatory reactions and insulin resistance. T‐Reg, naïve CD8 T cells, memory CD8 T cells, and memory CD4 T cells play important roles in maintaining immune tolerance and reducing inflammatory responses. It is possible to maintain blood sugar levels by preventing autoimmune‐mediated attacks on pancreatic beta cells [65–67]. At the disease level, analysis using CIBERSORT revealed that macrophages and monocytes were associated with the three diseases [68, 69]. The mechanisms of action of macrophages in AD are complex and vary at different stages of the disease. Following continuous stimulation by A*β* and tau proteins (Tau), different macrophages work synergistically to cause chronic, irreversible neuroinflammation, leading to the progression of AD. Patients with diabetes have increased inflammation due to the pancreatic islets, which can result in amyloid deposition, macrophage infiltration, and elevated levels of inflammatory cytokines and chemokines. For example, M1 macrophage polarization and pro‐inflammatory cytokine expression are increased in patients with diabetes, whereas M2 macrophage polarization and anti‐inflammatory cytokine expression are decreased. Dysregulation of M1 and M2 macrophages plays a major role in the development of some diabetic complications. Oxidative stress and inflammation heavily contribute to HTN, and the polarization of macrophages to the inflammatory phenotype plays a key role in these processes. HO‐1 acts as an inducible isoform of heme oxygenase (HO), and in macrophages, HO‐1 expression changes to an anti‐inflammatory phenotype, thereby improving vascular function and blood pressure. As for monocytes [70–72], monocyte‐derived inflammatory responses play a central role in the pathogenesis of T2DM. Furthermore, increased osmolality in subcutaneous tissues caused by long‐term high salt intake can activate monocyte tension‐responsive enhancer binding protein, which upregulates the expression of vascular endothelial growth factor C (VEGF‐C) and causes lymphatic vessel hyperplasia. Extensive hyperactivity of the subcutaneous lymphatic system can have a shunt effect on increased blood volume secondary to high salt intake to buffer the pressor response. However, blocking VEGF‐C secretion by monocyte mediators can induce salt‐sensitive HTN. Monocyte infiltration of different degrees and types can occur during lesion progression in HTN target organs. Monocytes are natural immune cells that effectively remove necrotic cells and debris. Many experimental studies on AD have shown that A*β* can recruit monocytes to the brain to limit amyloidosis in the brain.

There is a correlation between the key genes identified in this study and their enriched single‐cell types. For example, BGN is associated with angiogenesis, which, in turn, affects HTN in response to stress. Moreover, it is a biomarker of diabetic foot ulcers [73]. UBC, ELAVL1, and other key genes are related to macrophages and monocytes, which can influence the occurrence and development of diseases by affecting glucose and lipid metabolism.

Glucose and glucose metabolites can adversely alter protein structures through a nonenzymatic reaction known as saccharification, which is associated with the pathology of AD. T2DM can lead to abnormal inflammatory responses in multiple organs, including the brain, and insulin signaling is involved in amyloid breakdown. T2DM causes cerebrovascular disease, an important risk factor of AD. HTN affects blood supply by compromising the small blood vessels that provide oxygen to brain cells, and patients with HTN are prone to protein tangle and amyloid plaque formation in the brain, which can lead to the progression of AD. In addition, T2DM and HTN are often caused by insulin resistance or abnormal vascular endothelial function, which leads to various diseases.

To strengthen the validity of the predicted relationships between our eight shared key genes and the MAPK/mTOR pathways in the context of T2DM, AD, and HTN comorbidity, we have consolidated literature evidence for the key genes, with each showing consistent regulatory patterns toward the MAPK/mTOR pathways across T2DM, AD, and HTN, supporting their potential role as shared mediators of pathway dysregulation in comorbidity [74–80].

In T2DM, ACTN4 overexpression enhances MEK1/ERK phosphorylation in pancreatic *β*‐cells, while in HTN, it binds Raptor to potentiate mTOR‐S6K1 signaling and vascular smooth muscle proliferation—effects reversed by rapamycin, confirming a positive correlation between ACTN4 and MAPK/mTOR activity. As small leucine‐rich proteoglycans, BGN and PRELP synergistically regulate these pathways: their co‐expression upregulates p38 MAPK in AD hippocampal neurons, and overexpression activates mTOR in T2DM adipose tissue, indicating their positive regulatory role. TSFM links mitochondrial function to MAPK/mTOR signaling in both T2DM and HTN; its deficiency impairs mitochondrial respiration in diabetic cardiomyocytes, while its interaction with Rictor in hypertensive endothelial cells maintains mTORC2‐AKT‐eNOS signaling, underscoring its consistent regulatory function. UBC acts as a negative regulator via ubiquitination: It promotes K48‐linked mTOR degradation in AD neurons and interacts with ERK2 in T2DM to enhance its degradation, improving insulin secretion upon overexpression. ELAVL1 regulates MAPK/mTOR crosstalk by binding MEK1 mRNA to suppress translation in AD, and its downregulation enhances both MAPK and mTOR activity, increasing tau phosphorylation. In T2DM liver, ELAVL1 overexpression represses mTOR and gluconeogenic enzymes, lowering blood glucose. NRF1 negatively regulates both pathways under oxidative stress: It suppresses p38 MAPK via antioxidant induction in HTN and binds Raptor to inhibit mTORC1 and apoptosis in T2DM *β*‐cells. In addition, SUMO2 enhances MAPK/mTOR activity via sumoylation, modifying ERK2 to promote tau phosphorylation in AD and increasing mTOR sumoylation to stimulate S6K1 signaling and cell cycle progression in HTN, supporting a positive regulatory role. Together, these findings validate the coordinated involvement of these genes in MAPK/mTOR pathway dysregulation across the three comorbidities.

Although there have been many previous studies on the mechanisms of AD, HTN, and T2DM [81–85], there is a lack of research on the common pathogenic mechanisms between these three diseases, and few studies have provided a bioinformatics perspective. We found that there may be a common pathogenic mechanism underlying AD, HTN, and T2DM. Therefore, in this study, we identified shared genes between these three diseases using bioinformatics methods, and we analyzed the potentially enriched functions, pathways, and related cell types, which helped to further elucidate the molecular mechanisms underlying AD, HTN, and T2DM. However, this study was limited as we only preliminarily identified the eight key genes; experimental validation of these key genes is required in the future.

## 5. Conclusions

This study identified common biomarkers of AD, HTN, and T2DM using bioinformatic methods and found that the main biological functions and metabolic pathways shared by these three diseases are related to insulin resistance, vascular transport, and circulatory metabolism. In addition, endothelial cells, macrophages, and monocytes are associated with the occurrence of these three diseases, which may be mediated by key genes during pathological changes. Thus, these findings indicate that all three diseases affect the function of the associated cells through insulin resistance, vascular transport, and circulatory metabolism. The eight potential key genes identified in this study play important roles in this process.

In conclusion, this study provides possible targets for assessment methods for subsequent exploration of common pathogenic mechanisms between diseases. Combining these three diseases and identifying the key genes involved can improve precision medicine for patients with AD, HTN, and T2DM; however, such treatments require further confirmation through clinical trials.

## Ethics Statement

The authors have nothing to report.

## Disclosure

All the authors have read and approved the final version of the manuscript and submission to this journal.

## Conflicts of Interest

The author declares no conflicts of interest.

## Author Contributions

B.L. and H.Z. conceived the study and assisted with revising the manuscript. S.T. prepared and interpreted the data and wrote the first draft of the manuscript. W.Z. and Z.Z. participated in the data analysis and provided assistance with the article writing. Q.N., S.Z., and J.W. provided language assistance and proofread the manuscript. S.T., W.Z., and Z.Z. contributed equally to this work.

## Funding

This work was supported by National Natural Science Foundation of China (82474376), the National Key Research and Development Project (2023YFC3502903); the TCM Theory Inheritance and Innovation Project of the CACMS Innovation Fund (No. KYG‐202404) and the Fundamental Research Funds for the Central Public Welfare Research Institutes (ZXKT24002 and ZZ19‐XRZ‐119).

## Supporting information


**Supporting Information** Additional supporting information can be found online in the Supporting Information section. Figure S1: Expression profiles of ACTN4 in the control and disease groups. Figures S2, 23, and S4: Expression profiles of BGN, NRF1, and PRELLP in the control and disease groups, respectively.

## Data Availability

The datasets used and/or analyzed during the current study are available from the corresponding author on reasonable request.
