# Supplementary figures and images for: Analysis of the Genetic Comorbid Mechanisms of Type 2 Diabetes, Alzheimer′s Disease, and Hypertension Using Network Modularization
Source: Biomed Res Int. 2026 Feb 18;2026:8877510. doi: 10.1155/bmri/8877510 (PMC12914336; doi:10.1155/bmri/8877510)

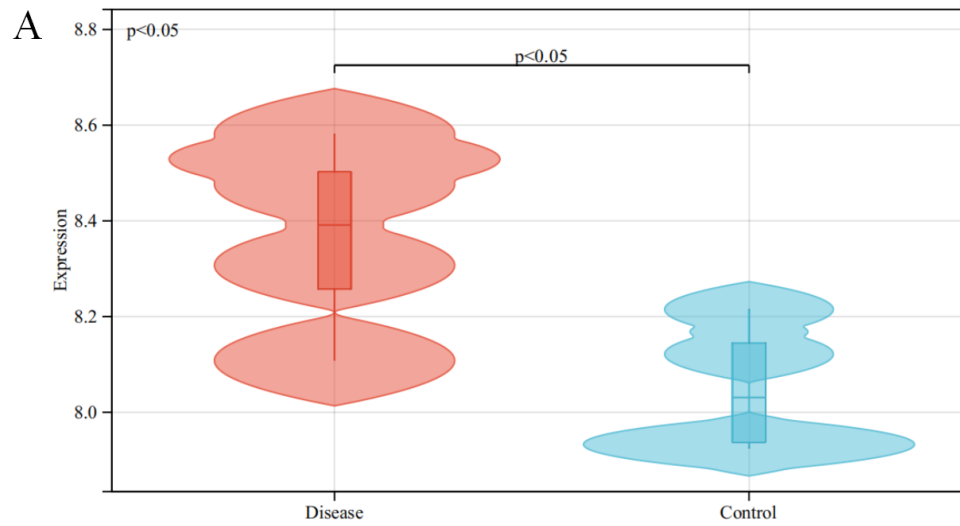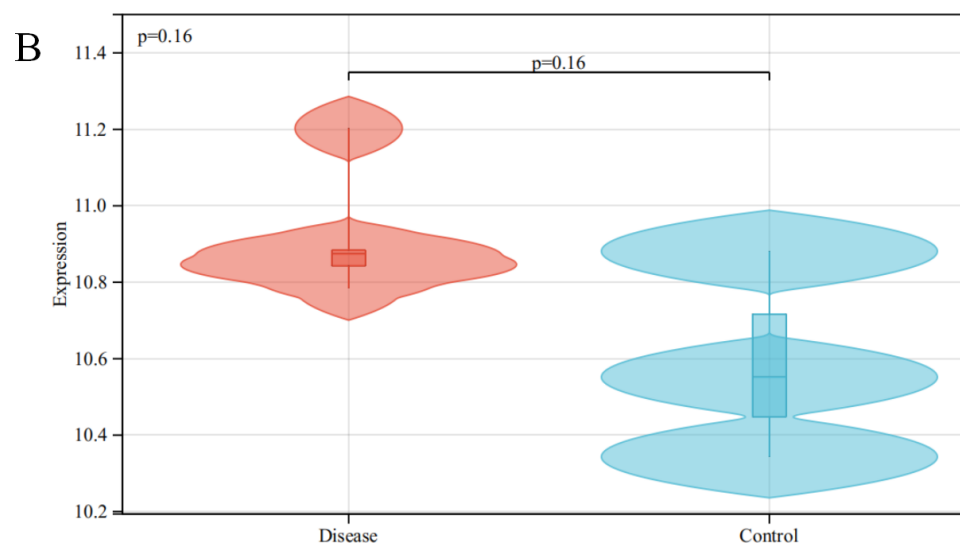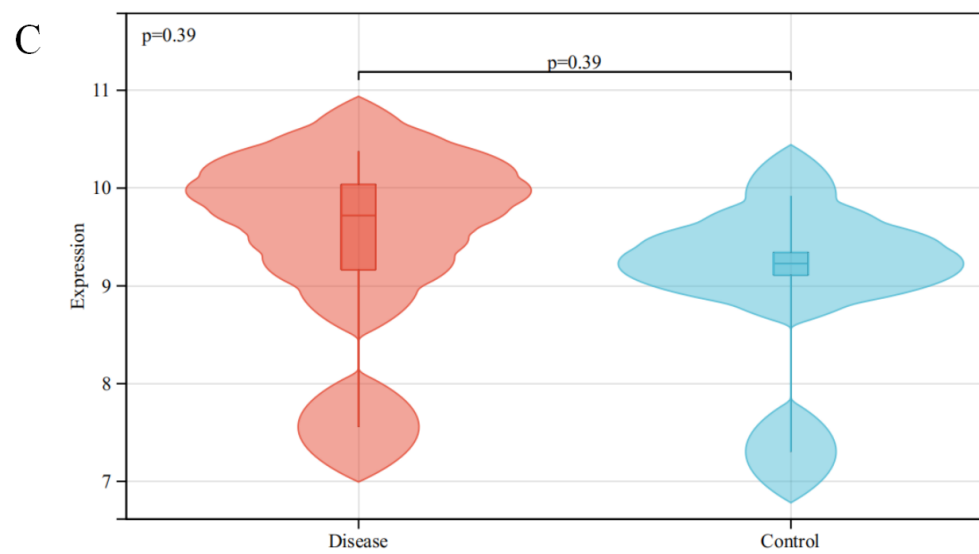

ACTN4

Supplement: Supplementary file 1 — Supporting Information Additional supporting information can be found online in the Supporting Information section. Figure S1: Expression profiles of ACTN4 in the control and disease groups. Figures S2, 23, and S4: Expression profiles of BGN, NRF1, and PRELLP in the control and disease groups, respectively. [file BMRI-2026-8877510-s001.zip › supplementary Fig.1-The expression profiles of ACTN4.pdf]

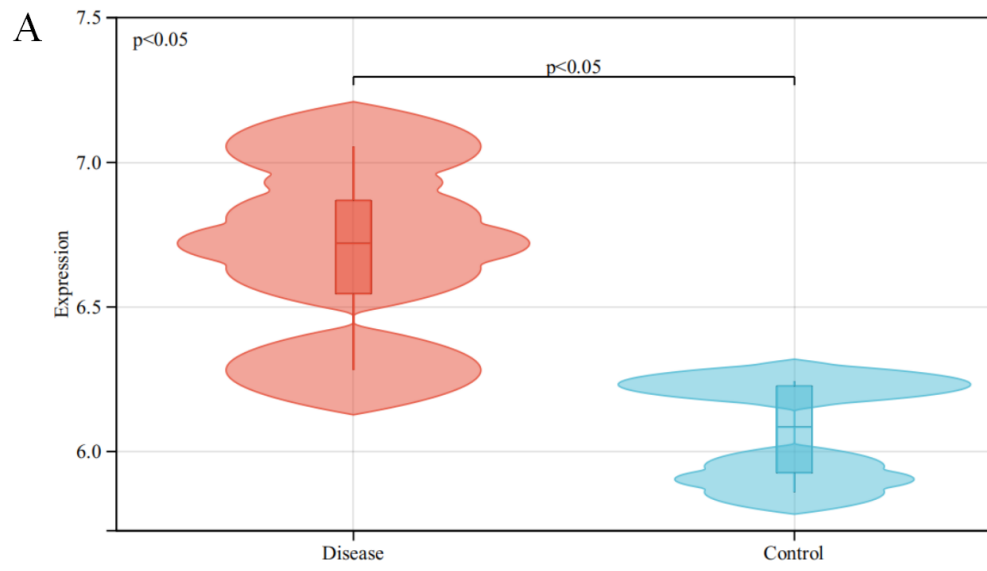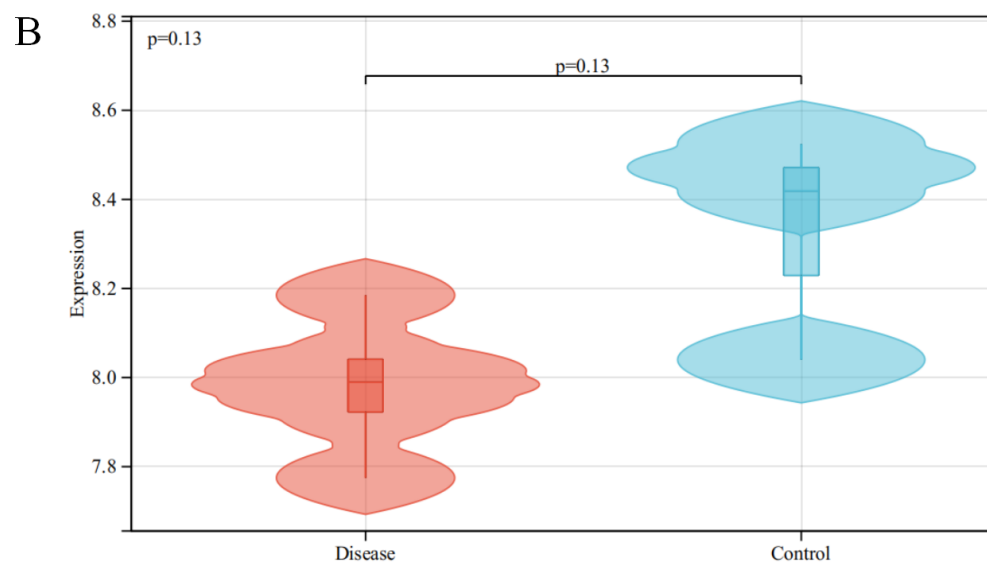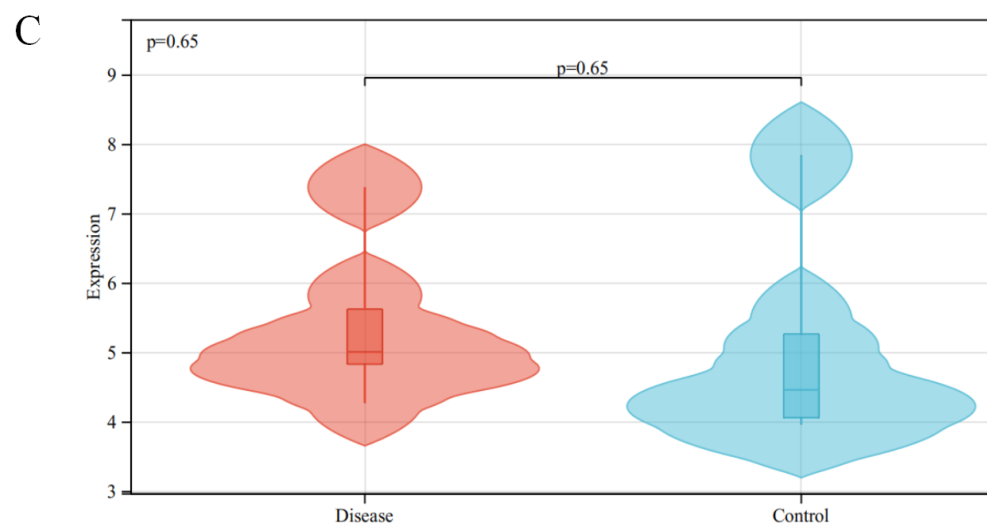

BGN

Supplement: Supplementary file 1 — Supporting Information Additional supporting information can be found online in the Supporting Information section. Figure S1: Expression profiles of ACTN4 in the control and disease groups. Figures S2, 23, and S4: Expression profiles of BGN, NRF1, and PRELLP in the control and disease groups, respectively. [file BMRI-2026-8877510-s001.zip › supplementary Fig.2-The expression profiles of BGN.pdf]

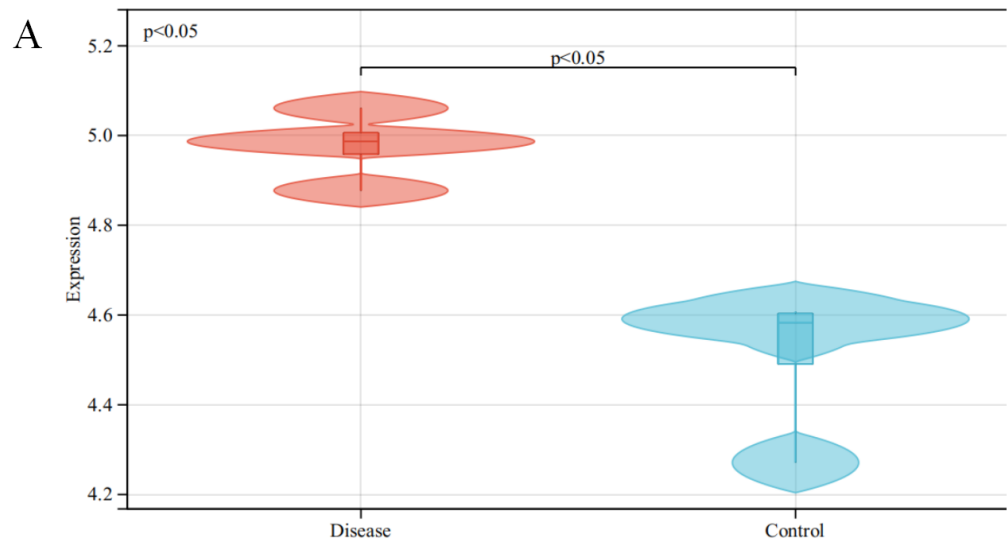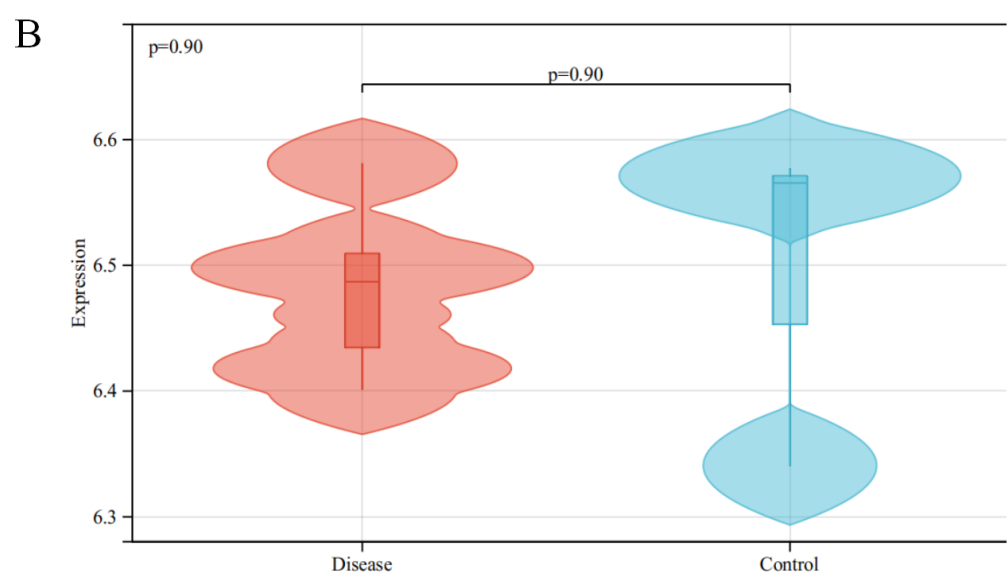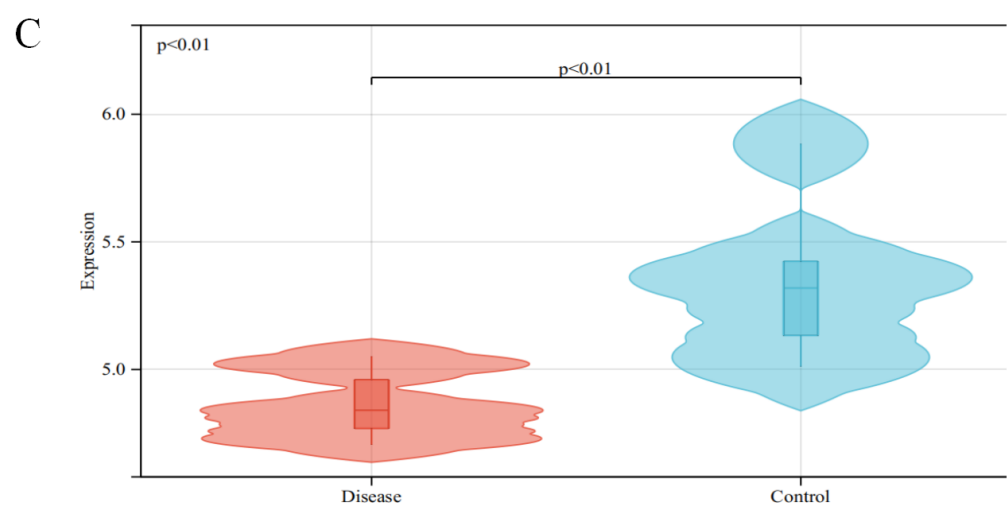

NRF1

Supplement: Supplementary file 1 — Supporting Information Additional supporting information can be found online in the Supporting Information section. Figure S1: Expression profiles of ACTN4 in the control and disease groups. Figures S2, 23, and S4: Expression profiles of BGN, NRF1, and PRELLP in the control and disease groups, respectively. [file BMRI-2026-8877510-s001.zip › supplementary Fig.3-The expression profiles of NRF1.pdf]

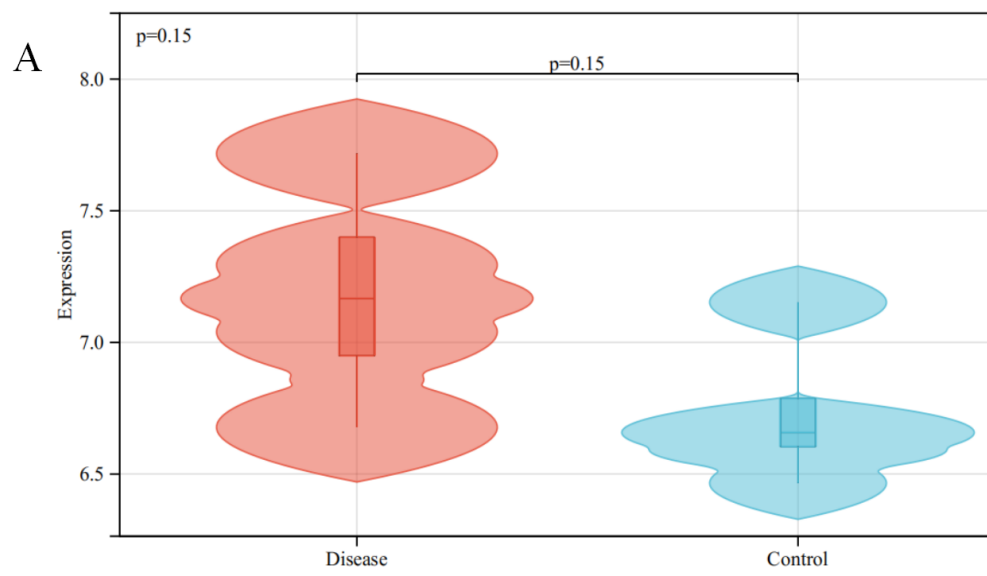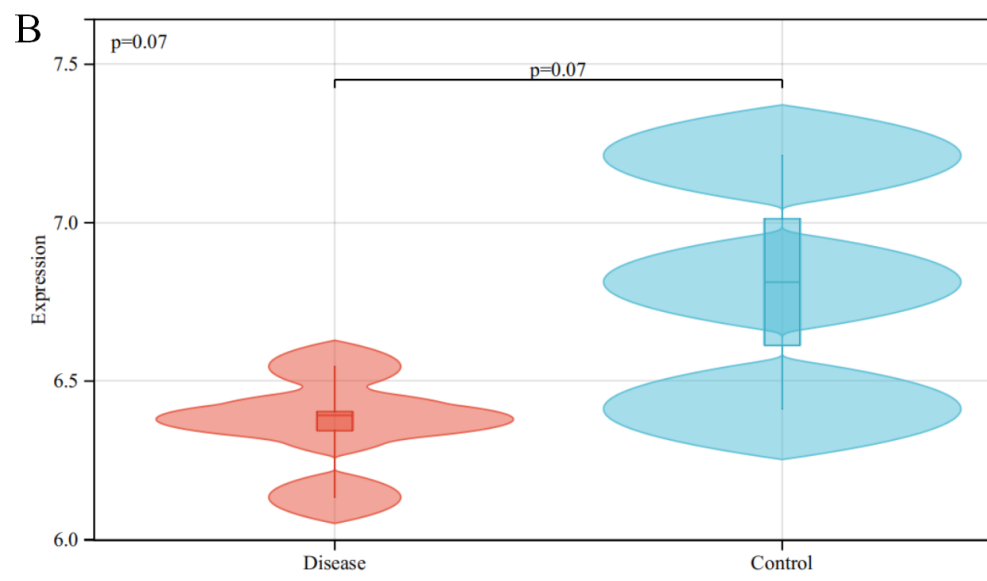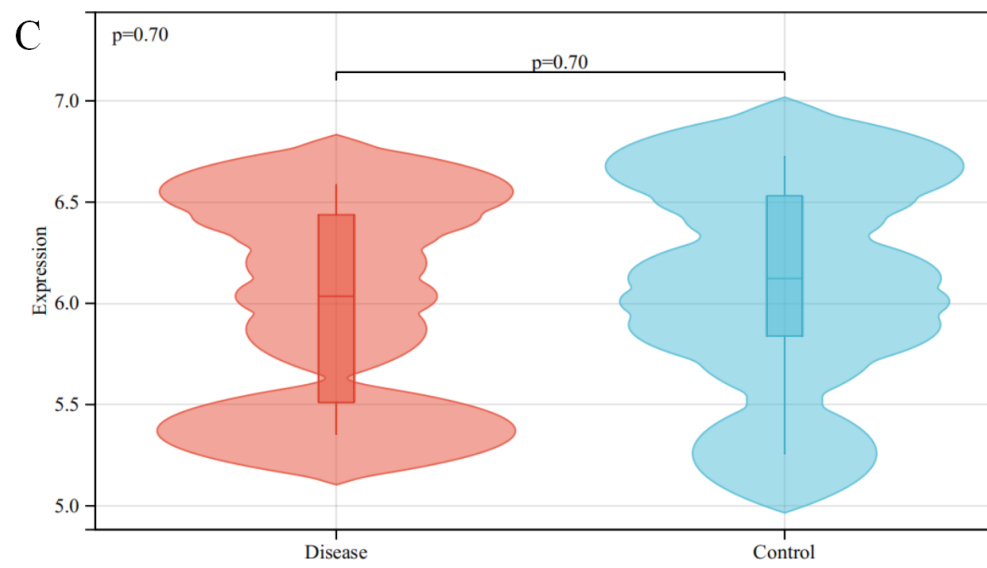

PRELP

Supplement: Supplementary file 1 — Supporting Information Additional supporting information can be found online in the Supporting Information section. Figure S1: Expression profiles of ACTN4 in the control and disease groups. Figures S2, 23, and S4: Expression profiles of BGN, NRF1, and PRELLP in the control and disease groups, respectively. [file BMRI-2026-8877510-s001.zip › supplementary Fig.4-The expression profiles of PRELP.pdf]
